# Implementing solid phase microextraction (SPME) as a tool to detect volatile compounds produced by giant pandas in the environment

**DOI:** 10.1371/journal.pone.0208618

**Published:** 2018-12-19

**Authors:** Abbey E. Wilson, Darrell L. Sparks, Katrina K. Knott, Scott Willard, Ashli Brown

**Affiliations:** 1 Department of Biochemistry, Molecular Biology, Entomology, and Plant Pathology, Mississippi State University, Mississippi State, Mississippi, United States of America; 2 Department of Conservation and Research, Memphis Zoological Society, Memphis, Tennessee, United States of America; 3 Mississippi State Chemical Laboratory, Mississippi State University, Mississippi State, Mississippi, United States of America; 4 Aquatic Systems and Environmental Health Unit, Resource Science Division, Missouri Department of Conservation, Central Regional Office and Conservation Research Center, Columbia, Missouri, United States of America; Universita degli Studi di Roma Tor Vergata, ITALY

## Abstract

Chemical cues are thought to play an important role in mate identification in the solitary giant panda (*Ailuropoda melanoleuca*). The goal of this study was to detect and identify volatile compounds present in the enclosure air of captive giant pandas. We hypothesized that a subset of compounds produced from breeding animals would be detected in environmental samples because highly volatile chemicals are likely to facilitate mate detection. Samples were collected from the enclosures of 8 giant pandas (*n* = 4 male, *n* = 4 female) during the Mar-June breeding season and the Aug-Jan non-breeding period from 2012–2015. Volatile compounds were captured by securing a solid phase micro extraction fiber approximately 3 meters above the ground within a panda enclosure for 6–12 hours. Compounds adsorbed onto the SPME fibers were analyzed by gas chromatography mass spectrometry. Thirty-three compounds were detected in at least 10% of all samples within individual and season and across all subjects within each season. Aromatic compounds made up 27.3% of the enclosure volatile profile, while 21.2% was made of cyclic aliphatic compounds and 51.5% of the enclosure profile was comprised of acyclic aliphatic compounds. Three compounds were likely to be present in male enclosures regardless of season, while Undecane, 4-methyl had a significant (*p*<0.05) predicted probability of being present in female enclosures. 3,3'-(1,1-Ethanediyl)bis(1H-indole) had a significant (*p*<0.05) probability of occurrence in male enclosures during the breeding season. Given the prevalence of these compounds, we suspect that these chemicals are important in giant panda communication. This novel sampling technique can detect volatile compounds produced by captive species and also may be a useful tool for detecting pheromones in free-ranging individuals.

## Introduction

Mammalian social and reproductive behavior is thought to be influenced by olfactory communication [1]. Mammals dispense chemical cues through various means to facilitate communication among individuals [2,3]. Vaporous compounds that initiate directed behaviors or developmental processes in an individual are considered pheromones. Typically, chemosensory compounds are volatile or semi-volatile molecules that are detected by chemosensory neurons in the vomeronasal organ and the main olfactory epithelium [3–5]. Due to the complex nature of mammalian social structures, it is expected that individuals distinguish important traits about conspecifics such as identity, territory, kinship, and reproductive status through chemical communication. Typical model species for mammalian pheromone research include mice [6] and rats [7]; however, recent studies have developed methods for the identification of pheromones in more exotic species such as the Asian elephant [8]. Compounds identified as potential pheromones in exotic mammalian species include short and medium chain carboxylic acids [9], steroids and long-chain fatty acids [10], and squalene [11]. It can be difficult to collect and identify pheromone candidates in mammalian species [3]; therefore, novel sampling techniques for the identification of pheromone candidates produced by exotic and often times vulnerable mammalian species are needed.

Developed in 1990, solid phase microextraction (SPME) is a relatively new technique for extracting chemical compounds from different matrix types [12,13]. SPME utilizes a chemically coated fiber to either absorb or adsorb volatile and semivolatile compounds, which can then be desorbed onto a gas chromatograph column and characterized by a detection system, such as a mass selective detector [14]. SPME was initially used for detecting pollutants in water samples [13], but has expanded to the field sampling of air samples [15,16]. SPME has been used to determine air quality [17–21], for the detection of odors in air [22–24], for on-site health monitoring [25–28], and pesticide identification in the atmosphere [29,30]. Furthermore, the detection of volatile compounds from the environment using SPME has been implemented to identify plant emissions [31–35] and insect pheromones [36–38]. The successful use of SPME to collect volatile compounds from the environment has been demonstrated across an array of subject areas; however, little research has been done to test the ability of SPME to detect mammalian pheromones in the environment. This tool has the potential to aid conservation efforts for threatened species through the non-invasive and real-time detection of pheromone candidates used for communication. By using SPME to collect volatile compounds from the environment, we will be able to identify particular chemical cues produced by species and may be able to gather information related to identity, age, and reproductive status of individuals.

The use of SPME as a tool to detect volatile compounds produced by giant pandas in captive enclosures is a novel sampling technique with applications for *in situ* conservation. The giant panda, *Ailuropoda melanoleuca*, is a vulnerable ursid in China [39] that relies on olfactory cues to communicate reproductive status [40–42], sex and age [10,40], kinship [43], and individuality [11,43–45]. A successful exchange of chemical cues is an important factor in the reproductive success of this species [46] as giant pandas are mono-estrus [39] with a brief window of sexual receptivity [39,47]. Previous studies have identified volatile compounds in giant panda urine [40–43] and ano-genital gland secretions [10,11,44,48]. These methods, therefore, require active collection of urine and secretions in addition to knowledge of where giant pandas excreted or emitted chemicals in the environment. These collections are difficult in wild studies due to low population numbers, decreased population density, and unknown locations of urine and secretion deposition. SPME may provide an alternative method for collecting volatile compounds produced by giant pandas. While this species was recently down-listed to vulnerable status due to an overall increase in population size [49], giant pandas require continued conservation and management aimed at facilitating scent communication.

This study aims to detect volatile compounds in the enclosure air of captive giant pandas. We hypothesized that a subset of compounds consistently produced from breeding animals will be detected in environmental samples as highly volatile chemicals could be candidate pheromones for mate detection. To test this hypothesis, our objectives were to (1) characterize the volatile profile of captive giant panda enclosure air using SPME air analysis and (2) identify differences in the enclosure air of male and female giant pandas during the breeding and non-breeding seasons. Furthermore, we aim to determine if SPME air analysis can detect similar compounds that have been identified in the urine of the same individuals in our previous study [50]. To our knowledge, this is the first study to use air analysis-solid phase microextraction in combination with gas chromatography mass spectrometry to investigate giant panda chemical communication in a captive setting.

## Materials and methods

### Animals

Male and female giant pandas housed at Memphis Zoo (MZ; Studbook #M466 and #F507), Zoo Atlanta (ZA; Studbook #M461 and #F452), Toronto Zoo (TZ; Studbook #M732 and #F676), and Edinburgh Zoo (EZ; Studbook #M564 and #F569) were the subjects of this study. At the time of the study, all giant pandas were at least 6 years of age and therefore, classified as sexually mature [39]. There were slight variations in diet at each institution with regard to bamboo species and supplements [51] and all bears received water *ad libitum*. Males and females at each institution were housed separately; however, exchange of olfactory, auditory, and visual cues were possible through the enclosures. Due to the reproductive management methods at each institution, male and female giant pandas may have had opportunities to enter the enclosure of the breeding partner during the spring breeding season. Additionally, male and female breeding introductions may have occurred; however, SPME sampling did not occur specifically during this time. Sampling continued that evening while giant pandas were housed in separate dens and/or the following day depending on the sampling protocol at each institution. This study was approved by the Memphis Zoological Society Institutional Animal Care and Use Committee (#2014–01). The collection of volatile compounds from the environment was conducted non-invasively by placing SPME fibers in the enclosure out of giant panda reach.

### Enclosure sampling

**Fig 1:**
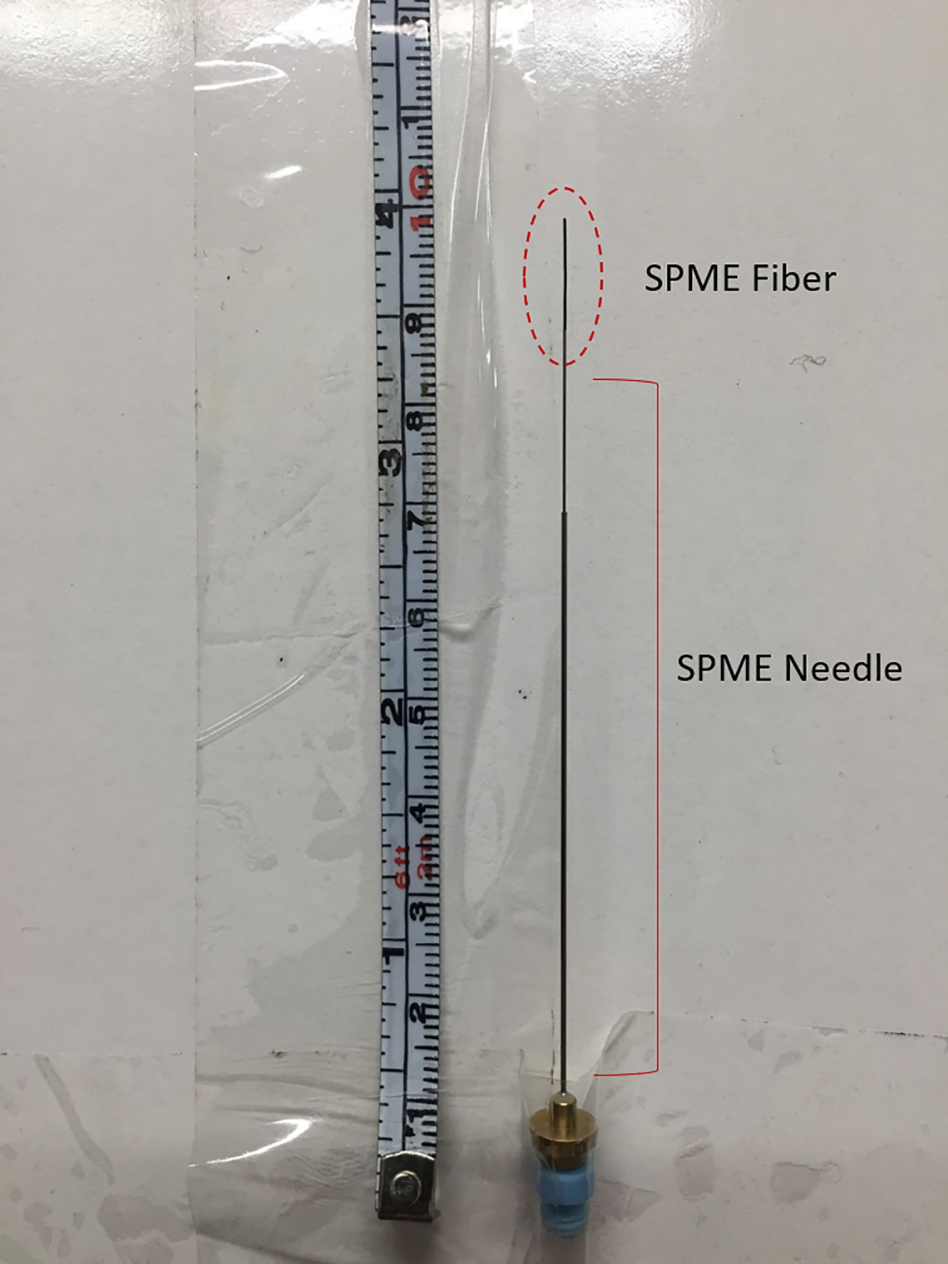
Solid phase microextraction fibers were mounted to the giant panda enclosure walls by securing the blue end of the SPME needle in a pocket made of clear tape. The fiber is exposed to collect volatile compounds in the environment.

Volatile compounds were extracted from the environment using a solid phase micro extraction (SPME) fiber with a carboxen/polydimethylsiloxane (CAR/PDMS) coating suitable for the extraction of gases and low molecular weight compounds (Supelco, Bellefonte, PA, USA). According to Sigma-Aldrich (supplier of Supelco SPME fibers), this coating is recommended for trace-level volatiles analysis (Sigma Aldrich, St. Louis, MO, USA). Several different types of fibers are available, including a polydimethylsiloxane/ divinylbenzene (PDMS/DVB)-coated fiber, which is appropriate for volatile polar analytes. However, there is no universal fiber and therefore, the selected fiber may have impacted the type of compounds this study was able to detect. The size of the giant panda exhibits ranged from 68m^2^ to 120m^2^ and the dens ranged from 7.67m^2^ to 16.23m^2^. The temperature of the giant panda enclosures was maintained between 17°C and 22°C at each institution throughout the sampling period. SPME fibers were secured to the walls of enclosures by placing the blue end of the fiber down in a pocket made of clear packing tape (Fig 1) and exposed to the environment for 6–12 hours during each sampling session over the course of 28 days. As a part of the routine maintenance procedure at each institution, the giant panda enclosures were cleaned of debris and sprayed down with water daily prior to securing the SPME fiber on the enclosure wall. The sampling session began when the giant panda was let into the enclosure area. The duration of the sampling period was dependent on animal cooperation and keeper availability at each institution. Upon retrieval, SPME fibers were immediately withdrawn into metal sheaths and kept in cool storage at 4°C. After the sampling period, SPME fibers were shipped overnight to Mississippi State University.

SPME fibers were used to collect volatile compounds in the exhibits and dens of female (*n* = 4) and male (*n* = 4) giant pandas during the spring breeding (March-June) and fall non-breeding (August-January) seasons from 2014–2016 (Table 1). Breeding season samples were collected either every two days or every day from Day -14 through Day 14 of the estrous cycle (28 days total), respective to when estrogen concentrations return to baseline (Day 0) in female urine (*n* = 57 female samples and *n* = 53 male samples). Non-breeding season samples were collected either every two days or every day for 28 days during anestrus (*n* = 33 female samples and *n* = 32 male samples). SPME sampling procedures were dependent on participating institutions and SPME fiber availability (Table 1). Memphis Zoo placed SPME fibers in giant panda exhibits and dens every 2 days in 2014 and in dens every night in 2015 for 28 days total (Day -14 through Day 14 of the estrous cycle) during the spring and for 28 days total during the fall, encompassing 2 breeding season collections and 1 non-breeding season collection. Zoo Atlanta placed SPME fibers in dens every other night in 2015 for 28 days, resulting in 1 breeding season collection and 1 non-breeding season collection. Because of shipping limitations, Toronto Zoo and Edinburgh Zoo performed an abbreviated SPME sampling protocol. SPME fibers were placed in enclosures on Day -14, Day 0, and Day 14 during the breeding season. Similar sampling was implemented during the non-breeding season, where SPME fibers were placed in enclosures on three separate days within a 28-day period to simulate the sampling method performed during the breeding season. While this abbreviated method does not provide the same number of samples as the other institutions, it still provides adequate sampling points to assess changes in the volatile profile across seasons as these dates correlate with the characteristic rise and fall of estrogen concentration in female giant panda urine during the estrous cycle[52] One SPME fiber was placed in a giant panda enclosure during the breeding season when no bear was present to aid in controlling for background chemicals collected from the environment.

**Table 1:** SPME enclosure samples collected for volatile analysis during 2014–2016 from Memphis Zoo, Zoo Atlanta, Toronto Zoo, and Edinburgh Zoo. Numbers in parenthesis indicate the number of samples analyzed each year (175 samples).

		Female		Male	
Institution	Sampling Procedure	Breeding	Non-breeding	Breeding	Non-breeding
Memphis Zoo	2014: exhibit and den every 2 days; 2015: dens every night.	2014(17) 2015(21)	2015(14)	2014(17) 2015(16)	2015(13)
Zoo Atlanta	Dens every other night.	2015(13)	2015(14)	2015(14)	2015(13)
Toronto Zoo	Exhibit on Day -14, Day 0, and Day 14.	2014(3) 2015(1)	2015(2)	2014(3) 2015(1)	2014(3)
Edinburgh Zoo	Exhibit on Day -14, Day 0, and Day 14.	2015(2)	2016(3)	2015(2)	2016(3)

### Analytical procedure

Prior to extraction, all SPME fibers had been conditioned at 250°C for a minimum of 30 minutes. Extracted volatile components adsorbed onto the fiber were analyzed using an Agilent 7890B GC (Agilent Technologies, Santa Clara, CA) with helium as a carrier gas passing through an Agilent ultra-inert DB-5MS column (30m x 0.25mm) coupled with the Agilent 5977A MSD modified from Dehnhard et al., 2005. In order to tentatively identify compounds, the ions present in the mass spectra and the relative ratios of the ions to each other in each chromatographic peak were required to match 80% of the known mass spectra of a compound present in the Wiley Registry 10th Edition/NIST 2012 Mass Spectral Library [44,48,53]. Similar to previous studies [10,43], tentatively identified compounds were not confirmed with known standards. Instrument blanks (no injection) were run approximately every 10 samples in addition to between sex and institution to prevent instrument contamination across samples.

### Data evaluation

We assumed that for a chemical to be important in interspecies communication of sex and reproductive status, the compound would occur relatively frequently in the samples available. Therefore, we chose to only consider volatile compounds that occurred in at least 10% of all samples within an animal and season [44]. Toronto Zoo and Edinburgh Zoo performed an abbreviated SPME sampling protocol, resulting in 3 or less samples collected from males and females during each season. Compounds occurring in these samples were not required to occur in at least 10% of the samples. Therefore, all compounds from the samples collected from Toronto Zoo and Edinburgh Zoo across both seasons during each year were included in the final criteria of occurring across all subjects. Although there is variation in the number of compounds present at each institution, volatile compounds related to sex and season should be maintained across individuals. Therefore, each individual giant panda was treated as a replicate and volatile compounds that occurred in at least one year from each institution and across all subjects (n = 4 male and n = 4 female) within one collection period were considered for statistical analysis. A collection period is defined as the time that environmental samples were collected for each sex. For example, compound A must occur in at least 10% of one female giant panda’s samples during the breeding season and across all females during the breeding season to be considered for statistical analysis; however, it may occur fewer times or not at all in female non-breeding season samples, male breeding season samples, or male non-breeding season samples. This method resulted in 38 compounds; however, 5 biologically irrelevant compounds (plasticizers and cleaning agents) were removed from the compound list. Additionally, compounds typically associated with clear packing tape (polypropylene and/or polyester) were not included in the analysis. Compounds identified in the control sample were included in the analysis, but are denoted in Table 2. These compounds were included because even though males and females at each institution were housed separately, exchange of olfactory cues were possible through the enclosures. Therefore, a total of 33 compounds (Table 2) were considered for statistical analysis.

**Table 2:** Tentatively identified compounds with a library match factor greater than or equal to 80 in giant panda enclosures. Thirty-three compounds occurred in at least 10% of all samples within individual and season and across all subjects within season. Compounds are organized by functional group chemical classification. The code for each compound is listed to the left of the compound name, which are used in the text and figures.

Code	Name and Classification	CAS Number
	**Aromatic**	
	***Ketone***	
C1	(Z)-3-Benzyl-5-(thiophen-3-ylmethylene)oxazolidine-2,4-dione^a^	2000425-67-1
	***Benzene derivative***	
C7	4-(4-Methoxybenzylideneamino)-5-(4-isopropylthiazol-2-yl)-4H-1,2,4-triazole-3-thiol^a^	2000627-24-9
C9	7-[(5-Mercapto-4-benzyl-1,2,4-triazol-3-yl)methoxy]-8-methyl-4-propyl-2H-1-benzopyran-2-one^a^	2000737-85-6
C11	Benzaldehyde^b^	100-52-7
C12	Benzene, (1-methylethyl)-	98-82-8
C13	Benzene, 1,2,4-trimethyl-	95-63-6
C14	Benzene, methyl-^b^	108-88-3
C20	Dicinnamylether	2000319-01-9
C33	3,3'-(1,1-Ethanediyl)bis(1H-indole)^b^	2000349-55-1
	**Aliphatic**	
	**Cyclic**	
	***Ketone***	
C6	2,5-Cyclohexadiene-1,4-dione, 2,6-bis(1,1-dimethylethyl)- ^a^	719-22-2
C17	Cyclopentane, methyl-^b^	96-37-7
C18	Cyclopropane, 1-(1-methylethenyl)-2-(2-methyl-1-propenyl)-, (1R-trans)-	80082-35-5
	***Alkene***	
C2	1,4-Cyclohexadiene, 1,6,6-trimethyl-3-methylene-^b^	94925-96-9
C4	2-Pinene^a^	80-56-8
C15	Bicyclo[2.2.1]hept-2-ene, 1,7,7-trimethyl-^a^	464-17-5
C16	Bicyclo[4.1.0]hept-3-ene-2-thiol, 3,7,7-trimethyl-, [1S-(1.alpha.,2.alpha.,6.alpha.)]- ^b^	88106-13-2
	**Acyclic**	
	***Ketone***	
C8	5-Hepten-2-one, 6-methyl-	110-93-0
	***Ester***	
C3	2-methyl-propanoic acid, 2,2-dimethyl-1-(1-methylethyl)-1,3-propanediyl ester^a^^,^^b^	6846-50-0
C10	Acetic acid, 2-ethylhexyl ester^a^^,^^b^	103-09-3
	***Aldehyde***	
C19	Decanal^a^^,^^b^	112-31-2
C22	Heptanal^b^	111-71-7
C23	Hexanal^a^^,^^b^	66-25-1
C25	Nonanal^a^^,^^b^	124-19-6
C26	Octanal^a^^,^^b^	124-13-0
	***Alkane***	
C5	2,2,6-trimethyloctane^a^	62016-28-8
C21	Dodecane^b^	112-40-3
C27	Octane, 2,6-dimethyl-	2051-30-1
C28	Octane, 6-ethyl-2-methyl-	62016-19-7
C29	Pentane, 2-methyl-	107-83-5
C30	Pentane, 3-methyl-^b^	96-14-0
C31	Undecane^b^	1120-21-4
C32	Undecane, 4-methyl-^b^	2980-69-0
	***Amine***	
C24	Hydrazine, 1,2-dimethyl-^a^^,^^b^	540-73-8

^a^Detected in enclosure while bear was absent

^b^Also detected in urine samples

### Statistical analysis

Binary logistic regression was used to test the hypothesis that the treatments (sex and season) have an effect on the presence and/or absence of specific volatile compounds in giant panda enclosures. Compounds taken into consideration for statistical analysis were present in 10% of samples and across all subjects (*n* = 4 male and *n* = 4 female). The dependent variable was the presence and/or absence of each volatile compound, while the independent variables were sex and season. Logistic regression models have been used for binary data in similar previous studies to determine if the presence and absence of volatile compounds are influenced by a series of categorical independent variables [54,55]. The presence and absence of compounds was used to measure differences relative to sex and season in giant panda enclosures. Each compound was considered an independently distributed response variable with a binomial distribution. If a compound was present, it was denoted with a value of 1. If a compound was not present in a given sample, it was given a value of 0. Each volatile compound was compared across season and sex; however, the presence of each compound was not compared to other compounds. The model can be used to calculate the predicted probability that a compound will be present when a particular sex (male/female) and/or season (breeding/non-breeding) was present [56]. The predicted probability is a value between 0 and 1, where 0 indicates no likelihood of occurring and 1 indicates 100% likelihood of a compound occurring in a given sex and/or season. A goodness-of-fit chi-square test was used to test for significance of the final model. All statistics were conducted using R statistical package with a level of significance set at 0.05 for all tests. Significant predicted probabilities for sex and breeding season treatment effects are shown following the compound name throughout the text.

## Results

### Characterization of compounds in enclosure

**Fig 2:**
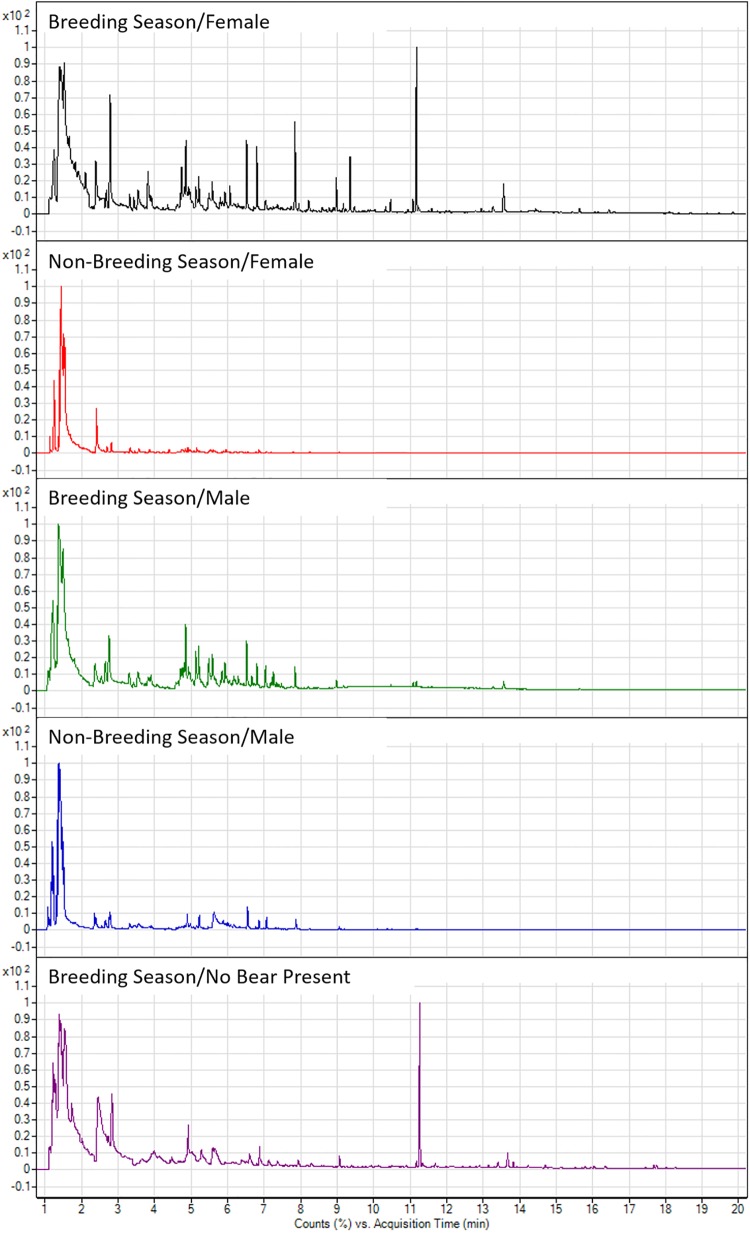
Total ion chromatograms from 1 to 20 minutes of giant panda enclosures (From top to bottom: Breeding season/female, non-breeding season/female, breeding season/male, non-breeding season/male, breeding season/no bear present).

Volatile compounds from 175 giant panda enclosure samples were analyzed in order to characterize the volatile profile of giant panda enclosures and investigate differences relative to season and sex with respect to the presence of specific compounds. Representative total ion chromatograms demonstrate qualitative differences between male and female giant panda enclosures during the breeding and non-breeding seasons (Fig 2). Female giant panda enclosures contained an average of 238.5 ± 121.3 (mean ± standard deviation) compounds during the breeding season (Fig 2a) and 223 ± 78.3 compounds during the non-breeding season (Fig 2b). Similarly, an average of 265.8 ± 132.8 compounds was detected in male giant panda enclosures during the breeding season (Fig 2c) compared to 222 ± 73.9 compounds detected during the non-breeding season (Fig 2d).

Thirty-three tentatively identified compounds with a match factor of 80 or greater occurred in at least 10% of all samples within each individual and season and across all subjects (*n* = 4 males and *n* = 4 females) within season (Table 2). The field air profile of giant panda enclosures consisted of aromatic and aliphatic compounds. These compounds were categorized into 10 classifications based on their structures and functional groups. Aromatic compounds made up 27.3% of the enclosure volatile profile, while 21.2% was made of cyclic aliphatic compounds and 51.5% of the enclosure profile was comprised of acyclic aliphatic compounds. The aromatic compounds consisted of ketones and benzene derivatives. The cyclic aliphatic compounds were comprised of ketones and alkenes, while the acyclic aliphatic compounds consisted of ketones, esters, aldehydes, alkanes, and amines. The percent presence of each compound sub-classification varied in giant panda enclosures. Benzene derivatives and acyclic aliphatic alkanes represented the majority of the field air profile at 24.2%, while aromatic ketones, acyclic aliphatic ketones, and acyclic aliphatic amines were the least present in the enclosure volatile profile (0.03%). Thirteen compounds were detected in giant panda enclosures when an animal was present and when an animal was absent (Table 2).

### Season and sex differences

**Fig 3:**
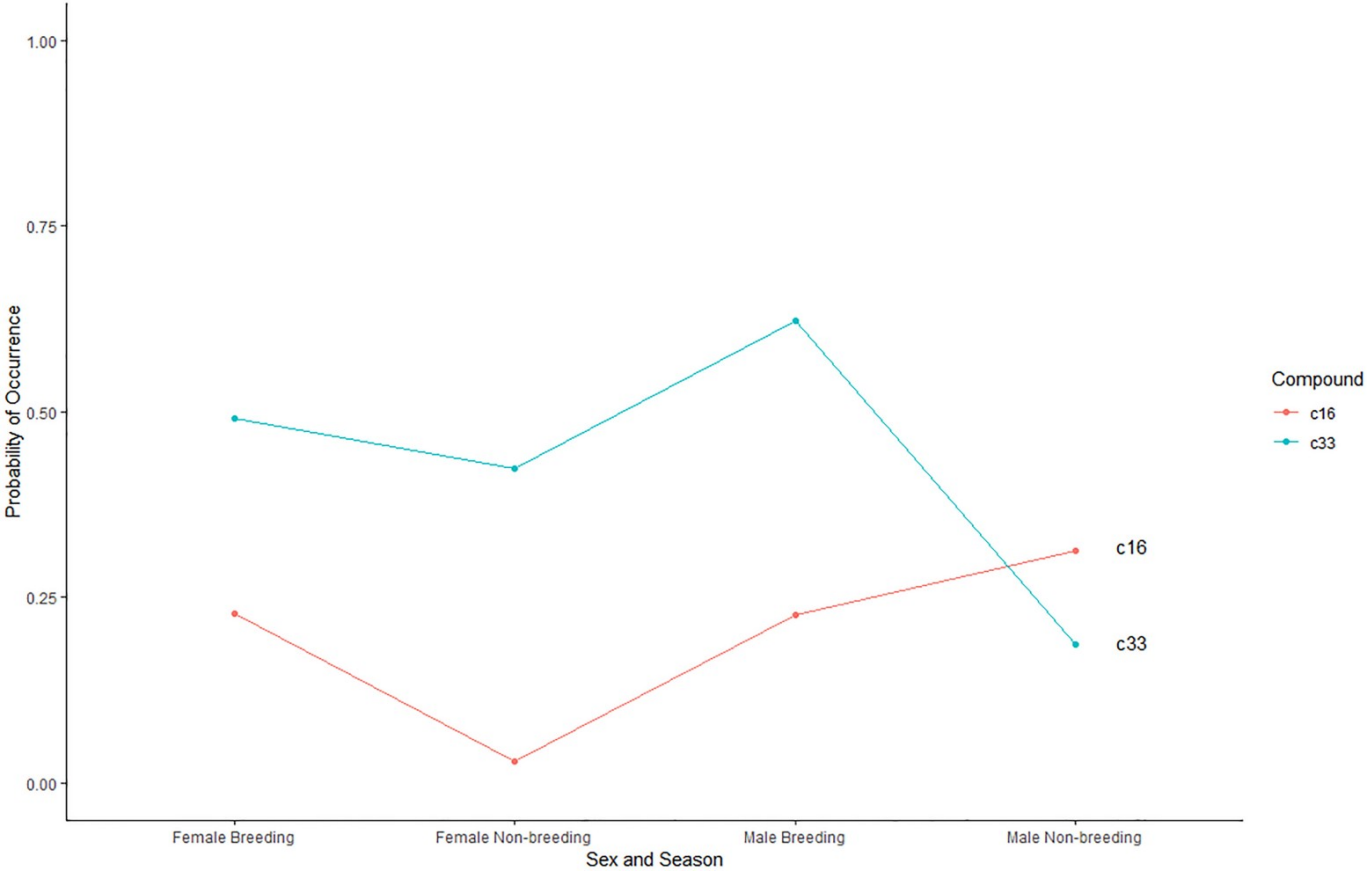
Significant probability of occurrence of C16: Bicyclo[4.1.0]hept-3-ene-2-thiol, 3,7,7-trimethyl-, [1S-(1.alpha.,2.alpha.,6.alpha.)]- and C33: 3,3'-(1,1-Ethanediyl)bis(1H-indole) across each collection period (*p*<0.05).

Binary logistic regression was performed to assess the effect of sex and season on the prevalence of 33 volatile compounds tentatively identified in giant panda enclosures. The full model included two independent variables (sex and season) and each compound was compared across 4 treatment groups, female breeding season (*n* = 57 samples), female non-breeding season (*n* = 33 samples) male breeding season (*n* = 53 samples), and male non-breeding season (*n* = 32 samples). The full model demonstrated statistically significant treatment effects (χ^2^(3) = 16.509, *p*<0.001, Nagelkerke R^2^ = 0.12) for sex and season interaction, sex only, and season only treatments on compound prevalence (S1 Table). C16 (0.3125) demonstrated a significantly (*p*<0.05) higher predicted probability in male enclosures during the non-breeding season, while C33 (0.6226) was significantly (*p*<0.05) more likely to be present in male enclosures during the breeding season (Fig 3).

**Fig 4:**
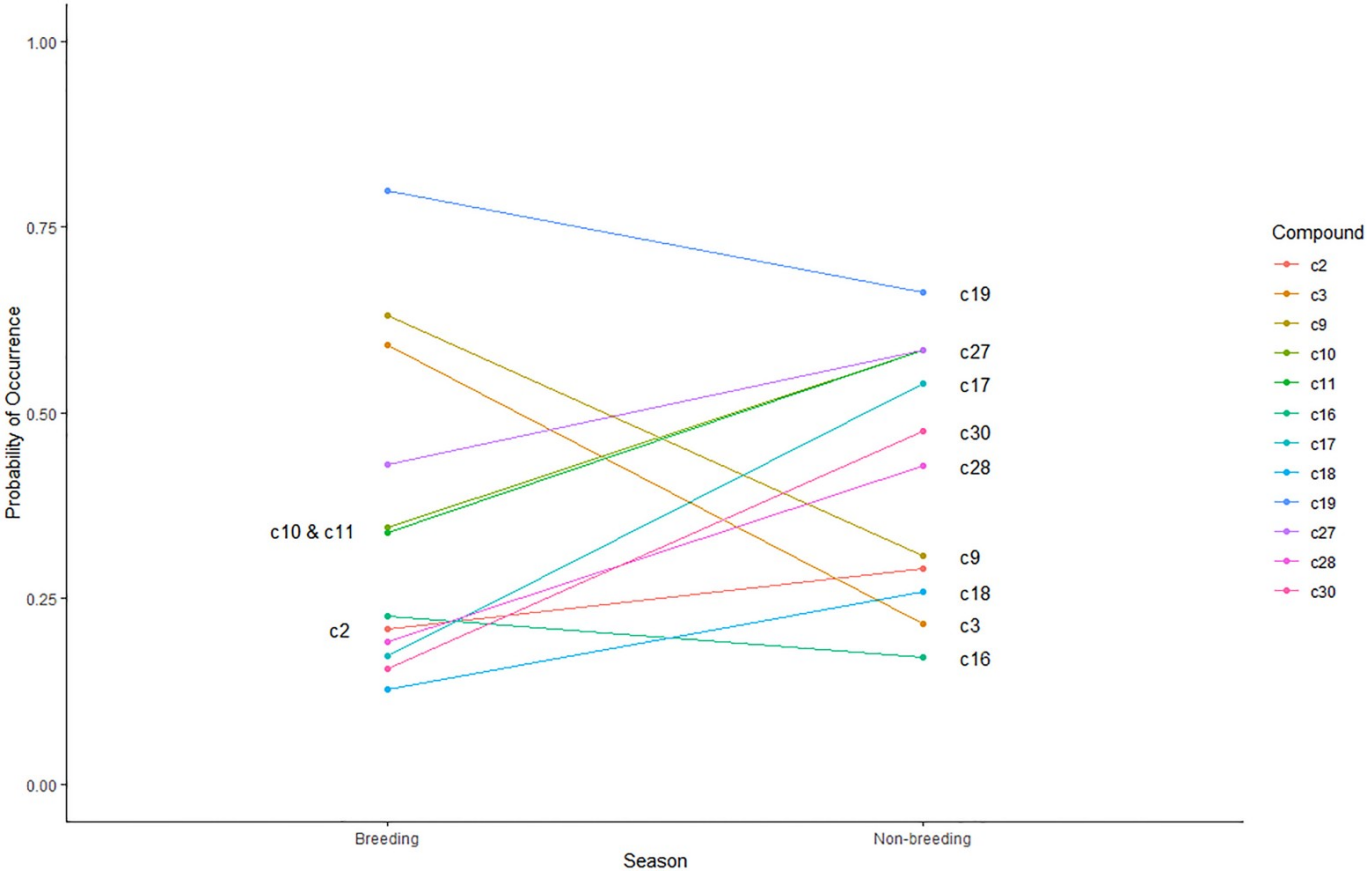
Significant probability of occurrence of C2: 1,4-Cyclohexadiene, 1,6,6-trimethyl-3-methylene-; C3: 2-methyl-propanoic acid, 2,2-dimethyl-1-(1-methylethyl)-1,3-propanediyl ester; C9: 7-[(5-Mercapto-4-benzyl-1,2,4-triazol-3-yl)methoxy]-8-methyl-4-propyl-2H-1-benzopyran-2-one; C10: Acetic acid, 2-ethylhexyl ester; C11: Benzaldehyde; C16: Bicyclo[4.1.0]hept-3-ene-2-thiol, 3,7,7-trimethyl-, [1S-(1.alpha.,2.alpha.,6.alpha.)]-; C17: Cyclopentane, methyl-; C18: Cyclopropane, 1-(1-methylethenyl)-2-(2-methyl-1-propenyl)-, (1R-trans)-; C19: Decanal; C27: Octane, 2,6-dimethyl-; C28: Octane, 6-ethyl-2-methyl-; C30: Pentane, 3-methyl-; across seasons (*p*<0.05).

**Fig 5:**
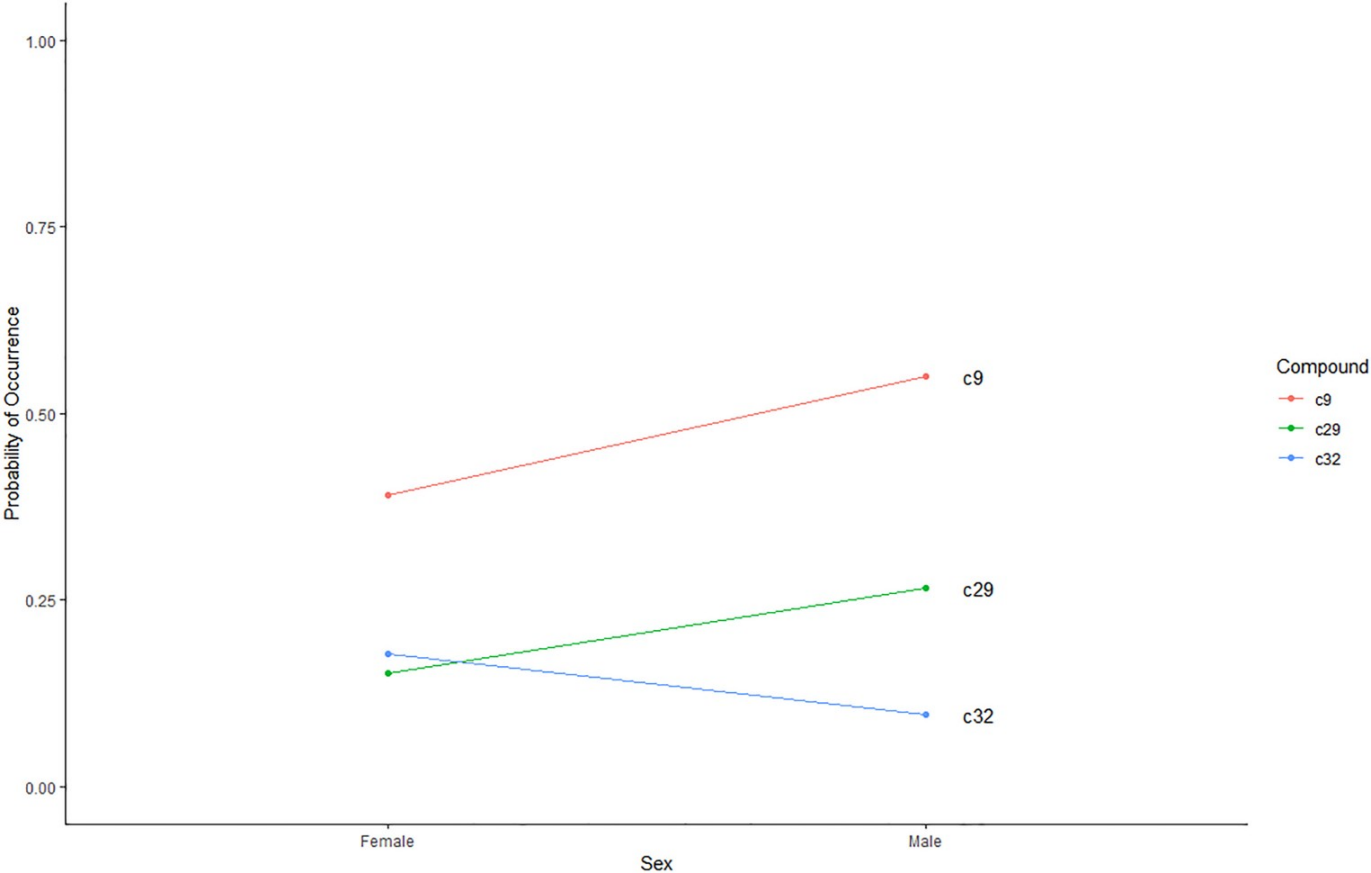
Significant probability of occurrence of C9: 7-[(5-Mercapto-4-benzyl-1,2,4-triazol-3-yl)methoxy]-8-methyl-4-propyl-2H-1-benzopyran-2-one; C29: Pentane, 2-methyl-; and C32: Undecane, 4-methyl- across sexes (*p*<0.05).

If no significant treatment interaction effect was demonstrated, the main effects of season and sex on compound prevalence were assessed separately. The predicted probability of 4 compounds being present significantly (*p*<0.05) increased during the breeding season, while the probability of 8 compounds being present significantly increased during the non-breeding season (Fig 4). C19 (0.7984), C9 (0.6317), C3 (0.5920), and C16 (0.2272) were significantly (*p*<0.05) more likely to be present during the breeding season (Fig 4). C2, C10, C11, C17, C18, C27, C28, and C30 (*p*<0.05) were significantly more likely to be present during the non-breeding season (Fig 4). The predicted probability of 2 compounds being present significantly (*p*<0.05) increased in male enclosures, C9 (0.5492) and C29 (0.2665). The predicted probability of C32 (0.1523) being present significantly (*p*<0.05) increased in female enclosures (Fig 5). No compounds were unique to a particular season and sex; however, C1was only present during the breeding season in both male and female enclosures. C7 was found in all collection periods except for female enclosures during the non-breeding season.

### Enclosure and urine comparison

Previous urinary volatile analysis completed with the same giant pandas in this study identified 146 volatile compounds of interest in giant panda urine [50]. Eighteen compounds were identified both in giant panda urine and in enclosures (Table 2). Out of these 18 compounds, 2 demonstrated statistically significant effects (*p*<0.05). The relative abundance per creatinine concentration (mg/mL)^-1^ of decanal was significantly (*p*<0.05) greater in male breeding season urine [50] and the probability of occurrence of this compound in the current study was significantly (*p*<0.05) greater in breeding season enclosures. Secondly, the relative abundance per creatinine concentration (mg/mL)^-1^ of pentane, 3-methyl- was significantly (*p*<0.05) greater in male urine [50] and the probability of occurrence of this compound in this study was significantly (*p*<0.05) greater in non-breeding season enclosures.

## Discussion

Qualitative differences were evident between enclosure air volatile profiles of male and female giant panda enclosures during the breeding and non-breeding seasons. Scent-marking behavior is prevalent during the spring breeding season months [39]; therefore, volatile compounds are likely to be greater in number and abundance to facilitate communication between conspecifics. Acyclic aliphatic compounds made up the majority of the enclosure air volatile profile (51.5%), while 27.3% of the air profile was comprised of aromatic compounds and the remaining 21.2% was comprised of cyclic aliphatic compounds. Each of these classifications contains varying structures that will influence the volatility of each compounds. Compounds that are highly volatile are typically chemical signals for short term communication [57]. Over half of the giant panda enclosure volatile profile consists of aliphatic acyclic compounds, which are open chain compounds (straight or branched) that do not contain any ring structures. Highly volatile compounds are likely to be detected by SPME of the enclosure air. Compounds that are highly volatile may move into the vapor phase more quickly and be spread into the environment, allowing for giant pandas to easily detect them. Aliphatic cyclic compounds and aromatic compounds have been detected in giant panda ano-genital secretions [10,11]. These compounds typically persist in the environment longer than aliphatic acyclic compounds, suggesting that these signals are used for long term communication.

The highly volatile compounds detected in the enclosure air are likely related to signaling sexual receptivity. Results from this study indicate that the presence of specific volatile compounds detected in giant panda enclosures are related to season and sex. Bicyclo[4.1.0]hept-3-ene-2-thiol, 3,7,7-trimethyl-, [1S-(1.alpha.,2.alpha.,6.alpha.)]- demonstrated a significantly higher predicted probability in male enclosures during the non-breeding season, while 3,3'-(1,1-Ethanediyl)bis(1H-indole) was significantly more likely to be predicted present in male enclosures during the breeding season. 7-[(5-Mercapto-4-benzyl-1,2,4-triazol-3-yl)methoxy]-8-methyl-4-propyl-2H-1-benzopyran-2-one showed a greater probability of occurrence in breeding season enclosures as well as in male enclosures. The only sex and season specific volatile compounds detected in enclosures were related to male giant pandas. Male giant pandas perform scent marking behavior more frequently and for a longer duration throughout the year compared to female giant pandas [39]. In addition, male giant pandas demonstrate physiological changes and sexual behaviors several months before the female is sexually receptive [58]. Based on this information, it is likely that males produce a greater number of compounds that are perhaps more easily detected by SPME. Compounds that are both season and sex dependent are likely related to specific pheromones as they convey detailed information to conspecifics. Furthermore, the presence and absence of particular compounds during the breeding and non-breeding season provide evidence for chemical signals related to reproductive status. Compounds that have an increased probability of occurrence during the breeding season likely indicate estrus status and sexual receptivity to conspecifics. Sexual differences in the enclosure volatile profile indicate male and female specific compounds that are likely used to facilitate mate identification. Future studies that investigate behavioral and physiological responses of giant pandas to specific compounds that vary across season and sex are needed in order to confirm potential pheromones.

Information related to conspecific identification [45], sex and reproductive status [59,60], adulthood and age [61], and competition [62] is gathered from olfactory communication in giant pandas. However, little is known about if and how giant pandas are able to detect conspecifics at far distances. Compounds deposited in giant panda secretions and urine are likely to volatilize into the environment. Therefore, we expected to see similarities between compounds detected in the environment and compounds identified in previous studies in ano-genital gland secretions and/or urine. The classes of compounds detected in urine by Liu et al. 2013 were similar to this study as both found ketones, benzene derivatives, and alkanes [40]. Specifically, this study detected dodecane and structurally similar compounds to indole, which have been previously identified in giant panda urine [40,43] Previous studies have identified compounds higher in molecular weight, such as steroids and long-chain fatty acids [10] and squalene [11] in giant panda ano-genital gland secretions. This study specifically detected dodecane, undecane, and benzaldehyde in giant panda enclosures in addition to structurally similar compounds such as octane and pinene, which were also found in giant panda ano-genital gland secretions [44]. Furthermore, previous studies have identified several ketones [44] and aldehydes [10,48] in the ano-genital gland secretions of giant pandas and these classes of compounds were also detected by SPME of the enclosure air. Depending on the structure and solubility of the compound, environmental factors such as temperature, pressure, and pH can either increase or decrease the volatilization rate and thus influence the chemical signal [57]. These factors may influence the differences noted between the enclosure profile and urine or ano-genital gland volatile profiles. Furthermore, larger compounds will persist longer in the environment as these compounds tend to be less volatile, while smaller compounds are likely to volatilize more quickly. Sampling for 6–12 hours allowed for highly volatile compounds as well as less volatile compounds with various molecular weights to be adsorbed onto the SPME fiber.

Thirteen compounds were identified in the enclosure area while the bear was absent; however, it is difficult to treat this as a true control because the enclosure area is not sealed. Therefore, odors and potential olfactory cues can be transferred across sampling areas. This technique may become more precise if the sampling area is closed off and does not allow air movement from other areas. However, this is not typically the case for large mammalian species housed in a captive environment. While some of the compounds identified in this study are not familiar, several others have been described in the literature. For example, benzaldehyde and pinene are well-known plant volatiles that influence insect reproductive behavior [63]. Therefore, some compounds detected by SPME air analysis may be attributed to the bamboo present in enclosures. Ketones and aldehydes are commonly found in mammalian secretions and have been described as potential pheromones [64]. Specifically, heptanal, decanal, and octanal have been identified in the hair of white-tailed deer [65]. This study also used a CAR/PDMS coated fiber to collect volatile compounds from the enclosure air of giant pandas because it is recommended for the extraction of low molecular weight compounds, gases, and trace-level volatiles (Sigma Aldrich, St. Louis, MO, USA). We believed low molecular weight compounds would be most likely to be detected by SPME air analysis as they will quickly volatilize into the environment. There are several types of SPME fibers available that are suited for a range of compounds. For example, a polydimethylsiloxane/divinylbenzene (PDMS/DVB)-coated fiber is appropriate for the extraction of volatile polar analytes, such as alcohols and amines. This study did not detect any alcohols and detected very few amine compounds. Therefore, the selected fiber may have impacted the type of compounds this study was able to detect as well as the range of molecular weights detected. However, there is no universal fiber and the only alternative would have been to conduct the experiments with multiple types of fibers, which was not practical in this setting.

Environmental effects on volatility may also affect the ability of giant pandas to detect chemical signals through their olfactory communication system. It is thought that free-ranging giant pandas will roam the bamboo forests in search of a mate during the breeding season [39]. It may be weeks before an individual locates a potential mate; however, chemical cues deposited in urine and scent marks may be providing vital information related to reproductive condition and sexual receptivity. With this in mind, organic compounds in giant panda urine must be volatile in order to reach the olfactory detection system in conspecifics, but also have a relatively high molecular weight so that the compound is able to persist over time. Depending on variation among environmental factors, this amount of time or rate of volatilization can vary and may ultimately influence not only the detection of pheromones by SPME fibers but also the detection of chemosensory molecules by giant pandas. By considering environmental information in combination with SPME detection of chemical signals in the environment, we may be able to address questions regarding the dispersion of volatile compounds in the wild and the detection ability of free-ranging giant pandas.

Applying SPME for detecting pheromones in mammalian species is a novel tool for management and conservation. This study has detected and identified volatile compounds in giant panda enclosures that are related to reproductive status and sexual receptivity. This research has the potential to provide information to management in order to facilitate a natural breeding environment for captive breeding programs through the identification of chemical cues that giant pandas use to communicate. Furthermore, this tool can also be used to gain a better understanding of how this species communicates across vast landscapes.

## Supporting information

S1 Table Binary logistic regression for testing the effects of sex and season on compound prevalence. Significant values are bolded (*p*<0.05).
(DOCX)
