# Supplementary material for: Implementing solid phase microextraction (SPME) as a tool to detect volatile compounds produced by giant pandas in the environment
Source: PLoS One. 2018 Dec 19;13(12):e0208618. doi: 10.1371/journal.pone.0208618 (PMC6300211; doi:10.1371/journal.pone.0208618)
Supplement: S1 Table — Significant values are bolded (p<0.05). (DOCX) [file pone.0208618.s001.docx]

Supplementary Table 1. Binary logistic regression for testing the effects of sex and season on compound prevalence. Significant values are bolded (p<0.05).

| **Variable** | **Estimate** | **Std. Error** | **z value** | **Pr(>\|z\|)** |
| --- | --- | --- | --- | --- |
| **C1** |  |  |  |  |
| (Intercept) | -1.3218 | 0.3249 | -4.0680 | **0.0000** |
| SEX | 0.3922 | 0.4456 | 0.8800 | 0.3790 |
| SEASON | -18.2443 | 1872.0334 | -0.0100 | 0.9920 |
| SEX :SEASON | -0.3922 | 2668.0581 | 0.0000 | 1.0000 |
| **C2** |  |  |  |  |
| (Intercept) | -1.4307 | 0.3356 | -4.2630 | **0.0000** |
| SEX | 0.2021 | 0.4694 | 0.4300 | 0.6669 |
| SEASON | 1.0000 | 0.4895 | 2.0430 | **0.0411** |
| SEX :SEASON | -1.2376 | 0.7433 | -1.6650 | 0.0959 |
| **C3** |  |  |  |  |
| (Intercept) | 0.2469 | 0.2669 | 0.9250 | 0.3551 |
| SEX | 0.2539 | 0.3893 | 0.6520 | 0.5143 |
| SEASON | -1.5590 | 0.5026 | -3.1020 | **0.0019** |
| SEX :SEASON | -0.2147 | 0.7181 | -0.2990 | 0.7650 |
| **C4** |  |  |  |  |
| (Intercept) | -0.4643 | 0.2721 | -1.7070 | 0.0879 |
| SEX | 0.7300 | 0.3884 | 1.8800 | 0.0602 |
| SEASON | 0.4037 | 0.4420 | 0.9130 | 0.3611 |
| SEX :SEASON | -0.0228 | 0.6409 | -0.0360 | 0.9717 |
| **C5** |  |  |  |  |
| (Intercept) | -0.7732 | 0.2850 | -2.7130 | **0.0067** |
| SEX | -0.1564 | 0.4174 | -0.3750 | 0.7079 |
| SEASON | -0.0597 | 0.4740 | -0.1260 | 0.8997 |
| SEX :SEASON | 0.2008 | 0.6805 | 0.2950 | 0.7679 |
| **C6** |  |  |  |  |
| (Intercept) | 1.2192 | 0.3157 | 3.8620 | **0.0001** |
| SEX | 0.2394 | 0.4721 | 0.5070 | 0.6121 |
| SEASON | 0.7618 | 0.6198 | 1.2290 | 0.2190 |
| SEX :SEASON | -0.5340 | 0.8628 | -0.6190 | 0.5360 |
| **C7** |  |  |  |  |
| (Intercept) | 0.2469 | 0.2669 | 0.9250 | 0.3550 |
| SEX | 0.3351 | 0.3915 | 0.8560 | 0.3920 |
| SEASON | -18.8129 | 1135.4457 | -0.0170 | 0.9870 |
| SEX :SEASON | 15.9623 | 1135.4459 | 0.0140 | 0.9890 |
| **C8** |  |  |  |  |
| (Intercept) | -0.3185 | 0.2683 | -1.1870 | 0.2350 |
| SEX | -0.0245 | 0.3869 | -0.0630 | 0.9500 |
| SEASON | 0.5008 | 0.4407 | 1.1360 | 0.2560 |
| SEX :SEASON | -0.8045 | 0.6407 | -1.2560 | 0.2090 |
| **C9** |  |  |  |  |
| (Intercept) | 0.0351 | 0.2650 | 0.1320 | 0.8946 |
| SEX | 1.0888 | 0.4149 | 2.6250 | **0.0087** |
| SEASON | -1.0159 | 0.4722 | -2.1510 | **0.0314** |
| SEX :SEASON | -0.7546 | 0.6808 | -1.1090 | 0.2676 |
| **C10** |  |  |  |  |
| (Intercept) | -0.6152 | 0.2775 | -2.2170 | **0.0267** |
| SEX | -0.0498 | 0.4014 | -0.1240 | 0.9013 |
| SEASON | 1.0460 | 0.4516 | 2.3160 | **0.0206** |
| SEX :SEASON | -0.1297 | 0.6443 | -0.2010 | 0.8405 |
| **C11** |  |  |  |  |
| (Intercept) | -1.0296 | 0.3008 | -3.4230 | **0.0006** |
| SEX | 0.6867 | 0.4101 | 1.6740 | 0.0941 |
| SEASON | 1.4604 | 0.4663 | 3.1320 | **0.0017** |
| SEX :SEASON | -0.8661 | 0.6497 | -1.3330 | 0.1825 |
| **C12** |  |  |  |  |
| (Intercept) | -0.6152 | 0.2775 | -2.2170 | **0.0267** |
| SEX | -0.4093 | 0.4172 | -0.9810 | 0.3266 |
| SEASON | -0.3656 | 0.4794 | -0.7630 | 0.4456 |
| SEX :SEASON | 0.2915 | 0.7025 | 0.4150 | 0.6782 |
| **C13** |  |  |  |  |
| (Intercept) | -1.2192 | 0.3157 | -3.8620 | **0.0001** |
| SEX | -0.2394 | 0.4721 | -0.5070 | 0.6121 |
| SEASON | 0.5261 | 0.4858 | 1.0830 | 0.2788 |
| SEX :SEASON | -0.7539 | 0.7722 | -0.9760 | 0.3289 |
| **C14** |  |  |  |  |
| (Intercept) | -1.4307 | 0.3356 | -4.2630 | **0.0000** |
| SEX | 0.5924 | 0.4496 | 1.3180 | 0.1880 |
| SEASON | 0.4499 | 0.5152 | 0.8730 | 0.3830 |
| SEX :SEASON | 0.2632 | 0.6931 | 0.3800 | 0.7040 |
| **C15** |  |  |  |  |
| (Intercept) | -0.0351 | 0.2650 | -0.1320 | 0.8950 |
| SEX | 0.3008 | 0.3834 | 0.7850 | 0.4330 |
| SEASON | 0.7282 | 0.4545 | 1.6020 | 0.1090 |
| SEX :SEASON | -0.2055 | 0.6549 | -0.3140 | 0.7540 |
| **C16** |  |  |  |  |
| (Intercept) | -1.2192 | 0.3157 | -3.8620 | **0.0001** |
| SEX | -0.0094 | 0.4554 | -0.0210 | 0.9835 |
| SEASON | -2.2465 | 1.0634 | -2.1120 | **0.0346** |
| SEX :SEASON | 2.6867 | 1.1765 | 2.2840 | **0.0224** |
| **C17** |  |  |  |  |
| (Intercept) | -1.5476 | 0.3482 | -4.4440 | **0.0000** |
| SEX | -0.0394 | 0.5050 | -0.0780 | 0.9378 |
| SEASON | 1.6082 | 0.4925 | 3.2650 | **0.0011** |
| SEX :SEASON | 0.2301 | 0.7095 | 0.3240 | 0.7457 |
| **C18** |  |  |  |  |
| (Intercept) | -1.9661 | 0.4036 | -4.8720 | **0.0000** |
| SEX | 0.0834 | 0.5722 | 0.1460 | 0.8842 |
| SEASON | 1.4065 | 0.5420 | 2.5950 | **0.0095** |
| SEX :SEASON | -1.2102 | 0.8339 | -1.4510 | 0.1467 |
| **C19** |  |  |  |  |
| (Intercept) | 1.6740 | 0.3632 | 4.6080 | **0.0000** |
| SEX | -0.5500 | 0.4836 | -1.1370 | 0.2554 |
| SEASON | -1.1144 | 0.5127 | -2.1730 | **0.0298** |
| SEX :SEASON | 0.7789 | 0.7143 | 1.0900 | 0.2756 |
| **C20** |  |  |  |  |
| (Intercept) | -2.5840 | 0.5185 | -4.9830 | **0.0000** |
| SEX | 0.5256 | 0.6759 | 0.7780 | 0.4370 |
| SEASON | 0.2814 | 0.7972 | 0.3530 | 0.7240 |
| SEX :SEASON | 0.5040 | 1.0032 | 0.5020 | 0.6150 |
| **C21** |  |  |  |  |
| (Intercept) | -0.2469 | 0.2669 | -0.9250 | 0.3550 |
| SEX | 0.2846 | 0.3831 | 0.7430 | 0.4580 |
| SEASON | 0.3075 | 0.4388 | 0.7010 | 0.4830 |
| SEX :SEASON | -0.4704 | 0.6273 | -0.7500 | 0.4530 |
| **C22** |  |  |  |  |
| (Intercept) | -0.1054 | 0.2653 | -0.3970 | 0.6910 |
| SEX | 0.1431 | 0.3819 | 0.3750 | 0.7080 |
| SEASON | -0.0770 | 0.4389 | -0.1750 | 0.8610 |
| SEX :SEASON | -0.6074 | 0.6377 | -0.9530 | 0.3410 |
| **C23** |  |  |  |  |
| (Intercept) | 0.2469 | 0.2669 | 0.9250 | 0.3551 |
| SEX | 0.0188 | 0.3848 | 0.0490 | 0.9609 |
| SEASON | -0.8065 | 0.4497 | -1.7930 | 0.0729 |
| SEX :SEASON | 0.5408 | 0.6356 | 0.8510 | 0.3949 |
| **C24** |  |  |  |  |
| (Intercept) | -0.3185 | 0.2683 | -1.1870 | 0.2350 |
| SEX | -0.2635 | 0.3924 | -0.6710 | 0.5020 |
| SEASON | -0.6624 | 0.4741 | -1.3970 | 0.1620 |
| SEX :SEASON | -0.2220 | 0.7155 | -0.3100 | 0.7560 |
| **C25** |  |  |  |  |
| (Intercept) | 0.9410 | 0.2948 | 3.1920 | **0.0014** |
| SEX | 0.3988 | 0.4490 | 0.8880 | 0.3745 |
| SEASON | -0.5102 | 0.4624 | -1.1030 | 0.2699 |
| SEX :SEASON | -0.5783 | 0.6749 | -0.8570 | 0.3916 |
| **C26** |  |  |  |  |
| (Intercept) | -0.0351 | 0.2650 | -0.1320 | 0.8950 |
| SEX | 0.2243 | 0.3826 | 0.5860 | 0.5580 |
| SEASON | 0.3405 | 0.4407 | 0.7730 | 0.4400 |
| SEX :SEASON | -0.7810 | 0.6304 | -1.2390 | 0.2150 |
| **C27** |  |  |  |  |
| (Intercept) | -0.6152 | 0.2775 | -2.2170 | **0.0267** |
| SEX | 0.6529 | 0.3905 | 1.6720 | 0.0946 |
| SEASON | 1.1748 | 0.4560 | 2.5760 | **0.0100** |
| SEX :SEASON | -1.0874 | 0.6395 | -1.7000 | 0.0891 |
| **C28** |  |  |  |  |
| (Intercept) | -1.6740 | 0.3632 | -4.6080 | **0.0000** |
| SEX | 0.4453 | 0.4896 | 0.9100 | 0.3630 |
| SEASON | 1.7346 | 0.5033 | 3.4470 | **0.0006** |
| SEX :SEASON | -1.1526 | 0.7068 | -1.6310 | 0.1029 |
| **C29** |  |  |  |  |
| (Intercept) | -1.9661 | 0.4036 | -4.8720 | **0.0000** |
| SEX | 1.0366 | 0.5058 | 2.0490 | **0.0404** |
| SEASON | 0.4620 | 0.6054 | 0.7630 | 0.4454 |
| SEX :SEASON | -0.6311 | 0.7913 | -0.7980 | 0.4251 |
| **C30** |  |  |  |  |
| (Intercept) | -1.8124 | 0.3813 | -4.7530 | **0.0000** |
| SEX | 0.2254 | 0.5284 | 0.4270 | 0.6697 |
| SEASON | 1.9947 | 0.5173 | 3.8560 | **0.0001** |
| SEX :SEASON | -0.7872 | 0.7287 | -1.0800 | 0.2800 |
| **C31** |  |  |  |  |
| (Intercept) | -0.3185 | 0.2683 | -1.1870 | 0.2350 |
| SEX | 0.2051 | 0.3843 | 0.5340 | 0.5940 |
| SEASON | -0.3747 | 0.4564 | -0.8210 | 0.4120 |
| SEX :SEASON | -0.0228 | 0.6461 | -0.0350 | 0.9720 |
| **C32** |  |  |  |  |
| (Intercept) | -1.5476 | 0.3483 | -4.4440 | **0.0000** |
| SEX | -1.6911 | 0.8006 | -2.1120 | **0.0346** |
| SEASON | 0.0435 | 0.5701 | 0.0760 | 0.9392 |
| SEX :SEASON | 1.5088 | 1.0400 | 1.4510 | 0.1469 |
| **C33** |  |  |  |  |
| (Intercept) | -0.0351 | 0.2650 | -0.1320 | 0.8946 |
| SEX | 0.5359 | 0.3879 | 1.3810 | 0.1672 |
| SEASON | -0.2703 | 0.4408 | -0.6130 | 0.5397 |
| SEX :SEASON | -1.6968 | 0.6926 | -2.4500 | **0.0143** |
